# The use of a novel urine biosensor platform for lung cancer detection during lung cancer screening

**DOI:** 10.1016/j.xjon.2026.101600

**Published:** 2026-01-30

**Authors:** Ory Wiesel, Tatiyana Suharev, Alaa Awad, Jannat Mukari, Adi Laser Azogui, Michal Mark Danieli

**Affiliations:** a Division of Thoracic and Esophageal Surgery, The Cardiovascular Institute, Baruch Padeh Tzafon Medical Center, Affiliated with the Azrieli Faculty of Medicine, Bar-Ilan University, Galilee, Israel; b EARLY OM, Natanya, Israel; c The Cardiovascular Institute, Baruch Padeh Tzafon Medical Center, Affiliated with the Azrieli Faculty of Medicine, Bar-Ilan University, Galilee, Israel

**Keywords:** volatile organic compounds, biosensor, lung cancer, lung cancer screening

## Abstract

**Objective:**

To evaluate the sensitivity and specificity of a novel biosensor platform using the olfactory system of rats to detect volatile organic compound signatures of lung cancer present in urine samples from patients eligible for lung cancer screening.

**Methods:**

Urine samples were collected from patients who met United States Preventive Services Task Force lung cancer screening criteria and underwent computed tomography scans, including individuals with proven lung cancer diagnosis and without lung cancer. Samples were analyzed using the biosensor platform, and biosensor responses were recorded. A dedicated machine learning algorithm assessed various behavioral parameters to estimate lung cancer risk. The system was validated against previously diagnosed cases and evaluated for sensitivity, specificity, positive predictive value, and negative predictive value.

**Results:**

A total of 238 patients were included. Among them, 144 (60%) were diagnosed with lung cancer, of whom 84 (58%) were male. Non–small cell lung cancer was diagnosed in 135 of 144 patients, with adenocarcinoma identified in 110 cases. Seven patients had small cell lung cancer, and 2 had large cell lung cancer. Stage distribution was 1 (stage 0), 89 (stage I), 12 (stage II), 18 (stage III), and 22 (stage IV). The biosensor platform achieved a positive predictive value of 91%, negative predictive value of 86%, sensitivity of 91%, and specificity of 86% (area under the curve = 0.86).

**Conclusions:**

In the era of precision medicine, the novel urine biosensor platform offers a simple, noninvasive approach to assist in identifying candidates for early lung cancer screening. Its integration could improve adherence to screening protocols and aid in the diagnosis and management of nonspecific pulmonary nodules.

## Graphical Abstract

**Figure:**
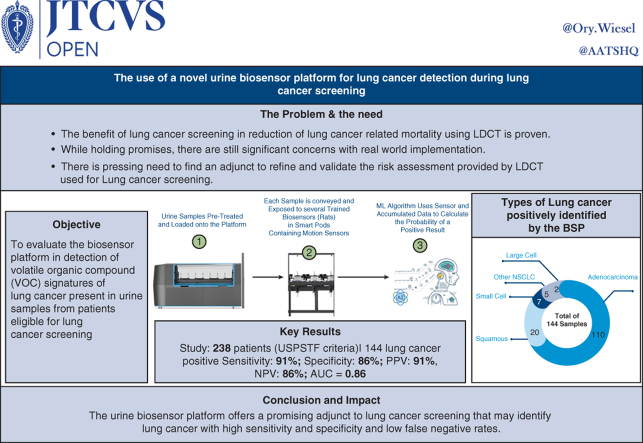

**Figure:**
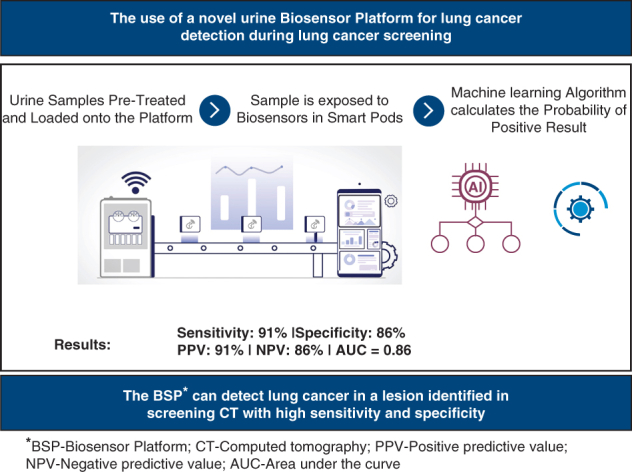
The novel urine biosensor platform for lung cancer detection during lung cancer screening.

**Central Message**

The urine biosensor platform might be useful along the diagnostic pathway of lesions identified in screening CT that may identify lung cancer with high sensitivity and specificity.

**Perspective**

Use of the urine biosensor platform in patients with lung cancer might improve diagnostic accuracy, refine current screening protocols, increase the low adherence to the current screening protocols, and might have an additional role in the decision-making process of detected pulmonary nodules and cancer recurrence.

Lung cancer is the leading cause of cancer-related mortality worldwide, projected to cost the lives of 127,070 patients in the United States by the end of 2023.^1^ Despite significant advances in the diagnosis and treatment of lung cancer, up to 70% of patients are still being diagnosed in advanced stages. Besides increased toxicity, decreased long-term survival, and worse patient outcomes, treatment at later stages creates additional costs, which increases overall health expenditure and overall burden on health care systems.^2^^,^^3^

Early detection of lung cancer has proved to save lives in multiple studies.^4^^,^^5^ Although 80% to 90% of patients diagnosed with stage I lung cancer achieve long-term survival, only 30% to 40% of those who are diagnosed in advanced stages can expect favorable outcomes.^2^^,^^3^

Besides primary cancer prevention, smoking reduction, and general public-awareness programs, cancer screening has proven to increase early cancer detection and diagnosis in multiple cancers. The National Lung Cancer Screening Trial laid the groundwork for multiple lung cancer–screening trials and subsequently established lung cancer–screening programs in the United States and across the globe using a series of low-dose computed tomography scans (LDCT).^5^^,^^6^ Although LDCT lung cancer–screening programs are associated with successful diagnosis of early cancers and reduction of lung cancer–related mortality, these tests are associated with disparities in adherence, overdiagnosis, overtreatment, low positive predictive value, and high cumulative false-positive rate when used sequentially.^7^ In addition, test inconvenience, duration of referral, test availability, and fear of ionizing radiation contributed to low test use within eligible populations.^5^

Besides adherence concerns and despite significant advances in screening protocols, improved technology, plasma biomarkers, 3-dimensional imaging, and artificial intelligence, there are still unanswered questions in the spectrum of early detection and lung cancer screening. Questions such as how to identify the ideal screening candidate across different populations at risk; what to do after LDCT scan results of determined and undetermined pulmonary nodules; and what to do in low-resource countries and underserved areas where screening is not available are actively being studied by many groups across the globe.

Given the impressive advancement in prevention and treatment options today, there is a pressing need to find a simple and cost-effective adjunct that will refine and validate the current screening pathways provided by using LDCT scans used for lung cancer screening. Biomarkers are heavily studied as adjuncts to current screening protocols with the hope of overcoming the need for a reliable, noninvasive, and reproducible lung cancer detection test that can augment diagnostic accuracy and the use of LDCT. The ideal biomarker will have the ability to reduce false-positive results and excessive diagnostic invasiveness in populations with a low prevalence of disease, and in populations with high prevalence of disease, an ideal biomarker will be able to reduce the rate of false-negative results.^8^

Recently, we described a novel biosensor platform that uses the extremely sensitive olfactory abilities of rats to detect volatile organic compounds from a urine sample of patients to provide an indication of lung cancer. Using rats as biosensors operating within a testing platform in a double-blind study, we validated the presence of lung cancer–specific volatile organic compound signatures in a urine sample, suggesting its potential benefit for lung cancer detection.^9^

The goal of the current study was to investigate the use of a biosensor platform as a useful test during the lung cancer diagnostic pathway of a patient with a lesion found during screening CT. Our primary objective was to test the urine samples of patients who met the criteria for lung cancer screening within our database on the basis of the current eligibility criteria for lung cancer screening United States Preventive Services Task Force (USPSTF). The secondary objective was to further validate the accuracy of our training and validation protocols on a larger cohort of samples with different prevalence of disease across multiple institutions and regions.

## Methods

### Patient Population

The study was designed as a retrospective, double-blind study. All patients were recruited between April 2021 and January 2023 by the study team from 4 different hospitals in Israel and 1 hospital in Korea, representing different lung cancer prevalence areas to ensure variability in the results. The study cohort included patients who were referred to thoracic surgery and oncology clinics at each participating hospital. All patients had to fulfill all the requirements of the USPSTF criteria for lung cancer screening (50-79 years old, history of at least 20 pack-years, current smokers, or those who quit less than 15 years ago). Patient demographics, lung cancer risk stratification, as well as smoking history and previous medical and oncologic history were recorded. Each patient underwent a CT scan of the chest during or before the first clinic encounter. Positive samples were identified by CT scan of the chest reports confirmed by biopsy or surgical specimen and assessed by a board-certified medical oncologist or thoracic surgeon. Negative samples were defined either by a negative radiology report reviewed by a board-certified radiologist or by negative biopsy or surgical specimen for lung cancer. Given the lack of a formal national lung cancer screening programs in Israel and Korea at the time of the patient recruitment, our patient cohort was chosen from referred clinic patients who inherently had the nature of greater prevalence of disease compared with the national average. All animal-related procedures were performed in strict accordance with the guidelines for the care and use of laboratory animals established by US the National Institutes of Health and the Animal Welfare Act and the Israeli Animal Welfare Act (1994)—Prevention of Cruelty and Abuse to Animals. Animal care conforms with Welfare Quality protocols as defined by the American Veterinary Medical Association, and in accordance with European legislation (Dir. 2010/63/EU), no animal was exposed to harmful conditions throughout this study. Patients were recruited during the first clinic encounter and upon suspicious radiology finding. Upon recruitment, urine samples were collected by the patient into a sterile container and aliquoted into coded 10-mL sterile polypropylene vacuum tubes. Samples were frozen at −20 °C within 6 hours after collection and transferred to EARLY OM central laboratory and were presented to the biosensor platform. The samples were tested following a strict double-blind methodology using an automated system without disclosing the identity of the samples or what they represented both to the biosensor (rat) and the operator. Only after obtaining the results did we compare the algorithm's findings with the subjects' clinical outcomes. We did not include patients who had received cancer treatment in the past year, those with immune-related conditions, and those who were pregnant. All tests were taken before the initiation of surgical or oncologic treatment, and treatment decision-making was not influenced by the test nor the test result.

### The Biosensor Platform

The biosensor platform combines biosensors, mechanical components, hardware, software, a cloud-based databased infrastructure, and a sophisticated machine learning algorithm capable of accurately discerning between positive and negative samples. The system comprises 2 principal components: the “operational area” and the “testing area.” The operational area contains the controller and (human) operator and is where the sample holders are loaded onto a conveyor belt. In the “testing area,” 3 identical smart pods for 3 biosensors are located. The samples are passed to the biosensors’ pods, which automatically open when positioned in front of the sniffing hatch, whereby the biosensor sniffs and reports its answer. Up to 3 samples can be assessed simultaneously by the platform, and each sample is tested by several separate and independent biosensors. Detailed specifications and characterization of the biosensor platform are thoroughly described in our previous manuscript.^9^

### Biosensor Performance

Performance parameters were recorded during all stages of training. All data related to the biosensors are collected during testing, including every sample interaction and their respective outcomes, which are automatically reported to our software. Machine learning algorithms compute the final sample results by using various measured parameters, including historical biosensors’ performance and success rates.

### Statistical Analysis

Diagnostic performance was assessed by calculating sensitivity, specificity, positive predictive value (PPV), and negative predictive value (NPV). Sensitivity was defined as the proportion of true positive cases correctly identified by the test and specificity as the proportion of true negative cases correctly identified. PPV and NPV were calculated to determine the probability that positive and negative test results, respectively, reflected the true disease status. All measures were reported with 95% CIs. Statistical analysis was performed using Jupyter Lab, version 4.4.1, and receiver operating characteristic curve analysis was conducted to evaluate overall test performance (area under the curve, AUC). The original contributions presented in the study are included in the article; further inquiries can be directed to the corresponding author.

This study was approved by each participating medical center institutional review board ethics committee as follows: in Israel-Sheba Tel-Hashomer (SMC-7655-20), Israel (September 21, 2020); Rambam Health Care Campus (RMB0276-210/0615-22), Israel (May 4, 2021); Shamir Medical Center (Asaf Harofe) (0293-20-ASF), Israel (January 20, 2021); Tazfon Medical Center (Poriya), Israel (0051-22), (September 19, 2022); and South Korea: Ewha Womans University Seoul Hospital) (August 10, 2020). Informed consent was obtained from all subjects involved in this study.

## Results

### Study Population

The study included a total of 238 urine samples from patients who met the USPSTF criteria for lung cancer. All patients underwent LDCT or CT scans of the chest. In total, 144 samples were from patients diagnosed with lung cancer on the basis of pathology results (lung cancer positive, ie, LCP), and 94 samples were from patients who were diagnosed as negative for lung cancer (lung cancer negative, ie, LCN) (Table 1).

**Table 1:** Patient characteristics

Study population	
Sex, n (%)	
Female	85 (36%)
Male	153 (64%)
CT findings, n (%)	
Negative	94 (39.5)
Female	25 (10.5)
Male	69 (29)
Positive	144 (60.5)
Female	60 (25.2)
Male	84 (35.3)
Age, y (average, range)	
Mean (SD)	64.4
Range	50-79
50-59, n (%)	72 (30.2)
Female	24 (10.1)
Male	48 (20.1)
60-69, n (%)	87 (36.5)
Female	32 (13.4)
Male	55 (23.1)
70-79, n (%)	79 (33.2)
Female	29 (12.2)
Male	50 (21)
Current smokers/stopped within 15 y (USPSTF criteria), n (%)	
Yes	238 (100)
Other types of cancers in LCN group, n (%)	
Breast	2 (2.1)
Esophageal	4 (4.25)
Multiple myeloma	1 (1.1)
Neoplasm of rib	1 (1.1)
Pancreas	3 (3.2)
Peritoneal carcinoma	1 (1.1)
Thymoma	2 (2.1)

*CT*, Computed tomography; *SD*, standard deviation; *USPSTF*, United States Preventive Special Task Force; *LCN*, lung cancer-negative.

### LCP Samples

**Figure 1:**
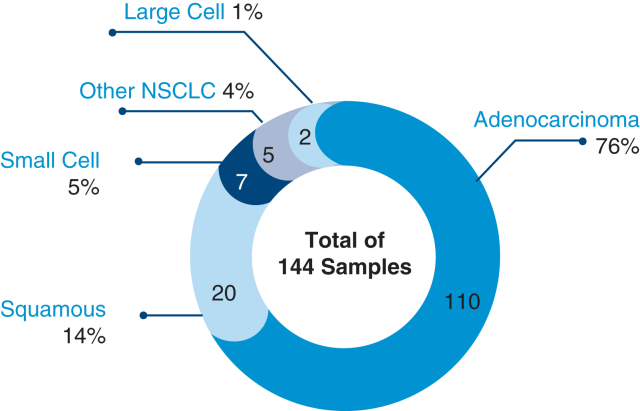
Lung cancer–positive (LCP) subtypes in the study population. Values are shown as n, (%). *NSCLC*, Non–small cell lung cancer.

Among the patients who were LCP, 84 (58%) were male. In total, 110 patients were identified to have lung adenocarcinoma and 20 lung squamous cell carcinoma. Small cell and large cell lung cancers were found in 7 and 2 patients, respectively. Five patients were identified solely as having non–small cell lung cancer (Figure 1).

### Stages

**Figure 2:**
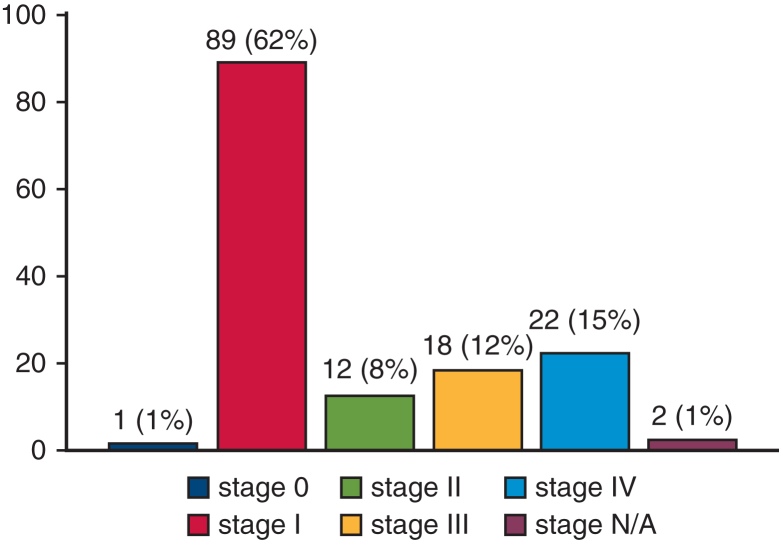
Number of patients in each lung cancer stages among the study population. Values are shown as n, (%). *N/A*, Not available.

One patient was diagnosed as having stage 0, 89 patients were at stage I, 12 as having stage II, 18 as having stage III, and 22 were diagnosed as having stage IV. In 2 patients the stage was not available (N/A) (Figure 2).

### LCN Samples

Among the 94 patients who were LCN, 69 (73%) were male. The LCN group included 14 patients who had a previous oncologic history of other types of cancer, 33 patients who were diagnosed with other lesions in their lungs, and 47 patients with negative scans. Table 1 shows the 14 LCN samples taken from patients who had diagnosis of other types of cancers at the time of recruitment. Of these 14 samples, 13 samples were recognized by the biosensors as negative for lung cancer true negative, whereas 1 one sample was further identified by the biosensors as positive to lung cancer (false positive) (Table 2).

**Table 2:** Biosensor platform performance in lung cancer–negative specimens

Study population	False positive	True negative	Specificity
Known previous cancer (14)	1	13	93%
Benign pulmonary nodules (33)	4	29	88%
Negative scan (61)	9	52	85%

The LCN group also included 33 patients who were diagnosed with lesions in their lungs. Of these 33 samples, 29 samples (88%) were identified as true negative, whereas 4 samples (12%) were identified as positive for lung cancer (false positive). Five of these 33 also had a previous oncologic history of other types of cancer. Four of them (80%) were accurately identified as LCN by the biosensor platform. In total, 61 of 94 LCN specimens had no apparent lesions in their lungs. Of these 61 samples, 52 (85%) were identified true negative, whereas 9 samples (15%) were identified as positive for lung cancer (false positive). In addition, 9 samples of these 61 lesion-free had previous oncologic history of other types of cancer. All of the 9 (100%) were accurately identified as LCN by the biosensor platform (Table 2).

### Diagnostic Accuracy

**Figure 3:**
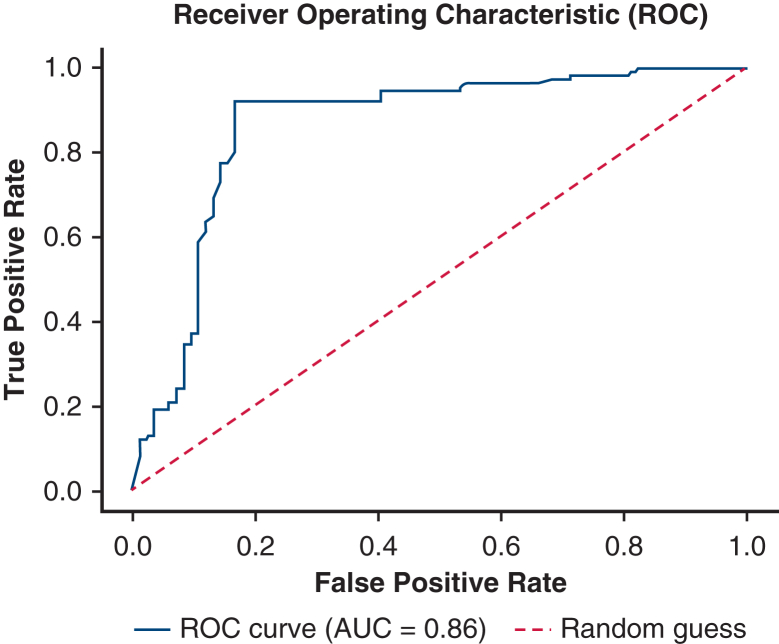
Receiver operative characteristic curve plotting true-positive versus false-positive rates of the biosensor platform in this study (AUC = 0.86). *AUC*, Area under the curve.

Overall, the biosensor platform achieved 91% sensitivity, 86% specificity, 91% PPV, and 86% NPV (Table 3). The receiver operating characteristic analysis showed that the biosensor platform had good discriminative ability to detect lung cancer in the investigated population with AUC = 0.86 (Figure 3).

**Table 3:** Biosensor platform performance in lung cancer–positive specimens

Lung cancer	Results			Predictive value
Positive	True positive = 131	False positive = 13	Sensitivity = 91%	Positive predictive value = 91%
Negative	True negative = 81	False negative = 13	Specificity = 86%	Negative predictive value = 86%

## Discussion

Several lung cancer–detection methods have been comprehensively studied over the past decades, and a number of molecular diagnostic tests have been developed on the basis of these studies. Despite significant advancements in recent years, early diagnosis of lung cancer continues to present challenges and engage clinicians and researchers.^10^

Emerging screening technologies and biomarkers are advancing quickly and are expected to gain broader acceptance after validation in well-designed clinical trials. Some of them hold significant potential to revolutionize lung cancer screening and diagnosis.^11^

A variety of biomarkers have been studied in patients with lung cancer, including those aimed at early disease detection, identifying minimal residual disease, and predicting relapse rates or treatment response.^12^ These biomarkers can be sourced from body fluids—such as serum, plasma, urine, saliva, and sputum—as well as from respiratory tract epithelial cells and exhaled breath.^11^

Current biomarkers have the potential to enhance the sensitivity and specificity of lung cancer–screening protocols, whereas other might help identify high-risk individuals after lung cancer resection and might offer effective follow-up strategies to minimize the risks associated with unnecessary invasive procedures^,^.^13^^,^^14^

Lung cancer early detection is increasingly supported by molecular biomarkers found in body fluids. Epigenetic changes such as DNA methylation and deregulated microRNAs are promising noninvasive biomarkers.^12^^,^^15^, ^16^, ^17^ Liquid biopsy techniques using circulating-free DNA (cfDNA) are another promising avenue for early lung cancer screening. cfDNA can reveal genetic and epigenetic abnormalities linked to early-stage lung cancer.^18^ Importantly, epigenetic markers from cfDNA may help stratify early-stage patients into high- and low-risk groups for recurrence and guide adjuvant therapy decisions.^18^ The exhaled breath of humans contains hundreds of volatile organic compounds, which can serve as a unique “fingerprint” to distinguish healthy individuals from those with cancer.^19^ The use of volatile organic compounds for early cancer detection is promising because of its noninvasive nature, low cost, and scalability and has proven not to be affected by the patient’s own diet or medications.^19^^,^^20^

Various breath-analysis methods have shown high sensitivity and specificity in lung cancer diagnosis. These include gas chromatography/mass spectrometry for detailed volatile organic compound profiling and electronic noses that detect breath patterns with promising diagnostic accuracy.^8^^,^^20^^,^^21^

In our previous study, we described in detail for the first time, to our knowledge, a novel biosensor testing platform for binary (positive/negative) volatile organic compounds signature recognition of lung cancer in a simple urine test by trained Long-Evans rats, with 93% sensitivity and 91% specificity.^9^ The biosensor platform leverages trained rodents as biosensors for detecting cancer-specific volatile organic compounds in urine samples, translating their conditioned behavioral cues, such as alert posture and focused sniffing into quantitative outputs via a standardized machine learning algorithm. Over a 4-month training period, the rodents undergo habituation, pretraining, active volatile organic compound conditioning, and performance validation using positive reinforcement, resulting in high sensitivity and specificity aligned with ground-truth diagnoses. Compared with canines, *Caenorhabditis elegans*, ants, and birds, which have been used in previous studies, rodents offer key advantages in scalability, cost-efficiency, regulatory control, and integration with automated detection systems while maintaining consistent performance under controlled laboratory conditions. This approach enables a reproducible, ethically compliant, and scalable solution for noninvasive cancer detection. Further, the simplicity of obtaining urine samples, their storage at standard freezing of −20 °C (used commonly at households and central laboratories), and their low maintenance costs, as well as the ease in housing and training the animals, their ability to perform in an automatic platform (such as the biosensor platform), their long attention span to perform several tests, and their relative long lifespans make the biosensor platform a potent and simple adjunct that might be useful even in countries and health care systems in which CT-based lung cancer screening programs are not commonly available, allowing for better selection of high-risk individuals who need to be scanned.

In this manuscript, we explore the potential benefit of the biosensor platform as a diagnostic tool along lung cancer screening pathway. Using the USPSTF criteria, we tested randomly 238 urine samples from eligible patients and found overall PPV of 91% with a NPV of 86% with a sensitivity of 91% and specificity of 86% (AUC = 0.86) for detection of lung cancer in our screened cohort.

Notably, the biosensor platform's false-negative rate in our cohort (5.4%) was lower than that reported for radiology-based models such as the Swensen model (9%) and Brock model (11%) in a previous study,^8^ as well as the false-negative rates reported for initial CT scans in lung cancer screening programs (ranging from 6.3% to 72.4% in different studies),^5^^,^^22^ false-negative rates of plasma biomarker assessments (ranging between 40% and 78.1% in different studies),^7^^,^^23^ and false-negative rates from tissue acquisition methods, with reported rates of 13% to 14%.^24^ These comparisons are particularly relevant for populations with an intermediate-to-high prevalence of lung cancer, such as those needed to be screened.

In order to overcome the inherent error of detection of a tested sample, each sample was tested blindly and randomly 6 times and by different biosensors to avoid selection, verification, and observer-type biases. The biosensor platform detected all types of lung cancer (non–small cell lung cancer as well small and large cell variants) and all stages of lung cancer. Interestingly, more than two thirds of the samples randomly tested were from patients with early stages of cancer (stage I, II), emphasizing the high sensitivity and the potential use of the biosensor platform in early lung cancer detection, specifically during lung cancer screening.

Using samples from 4 different hospitals within 1 country and 1 hospital in different continent represents a wide variety of patients from multiple regions, with different disease prevalence. Although one can argue that choosing 4 hospitals within the same country represent one disease prevalence region, we intentionally tested samples from different regions within the same country, representing potentially 4 different disease prevalence regions that represent diverse geographic and socioeconomic settings (urban high-density areas, industrial zones with elevated air pollution, and rural regions with limited preventive health services).

The Korean site provides data from a distinct East-Asian population with differing genetic and environmental backgrounds. We are now making an effort to expand our sample collection from various institutions across different countries and continents, including United Kingdom and the United States, to better represent wide variety of disease prevalence. Within each country, we are studying patients from different states, regions, and different health care systems. This approach aims to reduce both the false-positive rate in low-prevalence populations and the false-negative rate in high-prevalence populations with the hope to be able to improve the sensitivity and specificity of the biosensor platform in detecting different disease prevalence and across different populations in-order to optimize the platform for generalized use.

Our cohort had 94 lung cancer–negative samples representing 3 types of patients’ population that might be represented in typical lung cancer screening programs. A total of 14 patients were known to have history of previous cancer in whom the biosensor accurately identified the samples as negative for lung cancer in 13 patients with a specificity of 93%. A total of 33 patients had other lesions in their lung in whom the biosensor accurately identified the samples as negative for lung cancer in 29 patients with specificity of 88%. Finally, 61 samples were collected from patients with lesion-free negative scans; of them, 52 were labeled negative by the biosensor, resulting in true negative result of 85%. With the hope to better define populations at risk for further intervention, these data highlight the potential benefit of the biosensor platform as an adjunct for the current follow-up screening guidelines for undetermined pulmonary nodules and for better selection of those patients in whom we should perform an invasive test in conjunction with screening protocols. We are now conducting a follow-up study in endemic areas for non-neoplastic and infectious pulmonary nodules to improve our training protocols and the biosensor's detection ability in such regions.

### Future Perspective of the Biosensor Platform

**Figure 4:**
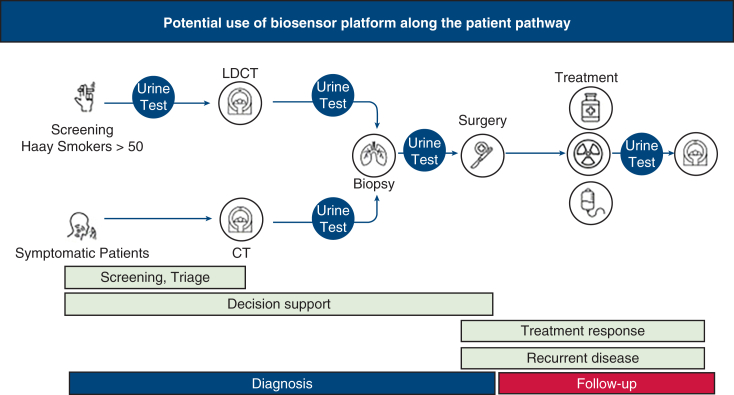
Potential use of biosensor platform along the patient pathway. *LDCT*, Low-dose computed tomography; *CT*, computed tomography.

Although not scientifically proven nor rigorously studied prospectively in randomized trials, volatile organic compound–based detection by biosensor platforms might not be limited only as a useful tool to be used during the diagnostic pathways of a lesion detected in a screening CT. Ongoing studies are undergoing in order to validate its simplicity and reproducibility during lung cancer screening in different populations and disease prevalence, as an adjunct for decision support, as monitoring adjunct for treatment response and detection of recurrence of lung cancer following surgery (Figure 4). We previously reported the potential benefit of the system in the decision making process of complex patients in whom no available tissue confirmation could be made, or multiple biopsies found to be negative in morbid patients.^25^ Although it has not yet been tested for quantitative or qualitative assessment, after curative resection and a negative test detection of volatile organic compounds in the urine during the patient's follow-up can mark an early relapse or progression of disease and might affect decision-making and treatment modification in a similar fashion like cfDNA. Finally, in some underserved areas, and in regions where lung cancer–screening programs using low-dose CT are limited for multiple reasons, biosensor-based platforms can be used for early detection and prescreening in high-risk individuals once validated.

Although promising, our study has several limitations. Our cohort was collected from patients from 5 different hospitals, 4 of which are within 1 country. This might pose selection bias, which is inherent in testing population at risk and might not represent real-world data in different disease prevalence regions. During the study enrollment period, there was no national screening protocol in our country; hence, we included patients who were referred to tertiary thoracic and oncology clinics and matched the USPSTF criteria. We acknowledge the fact that our patient cohort had greater pretest probability for lung cancer and were at greater risk for lung cancer compared with the national prevalence. This recruitment design was necessary initially to ensure inclusion of pathologically confirmed lung cancer cases for algorithm training and validation as well as evaluate the efficacy of the biosensor platform while maintaining adherence to established screening eligibility criteria.

An ideal study design would be a large prospective study of screened patients on the basis of national lung cancer screening protocols recruited from multiple nations and populations. Furthermore, we still need to prove the reproducibility of our technology and protocols in larger cohorts, across multiple populations, ethnicities, different disease stages, subtypes, and molecular profiles followed by validation in prospective large international cohort studies before it would be ready to be used in clinical setting and in multiple indications. Additional validation should be made in endemic infectious disease regions where pulmonary nodules are frequently being found in CT scans of the chest. Finally, we recruited our patients on the basis of the USPSTF criteria. Given the low adherence of screening programs, it is still undetermined whether this is the ideal criteria that should be used for biosensor-based protocols. Other high-risk groups such as those with environmental exposure, family history of lung cancer, high-risk occupational exposure, and genetically predisposed populations that are not identified in current age and smoking history-based screening protocols might be potential candidates for biosensor based protocols.

## Conclusions

In the era of precision medicine, the urine biosensor platform offers a promising noninvasive tool throughout the diagnostic pathway of patients with a lesion identified during lung cancer screening CT. It may identify lung cancer with high sensitivity and specificity and low false-negative rates. Use of the system might have additional role in the management of undetermined pulmonary nodules detected during lung cancer screening pathways. Further validation is needed to assess its implementation in clinical settings.

### Webcast

You can watch a Webcast of this AATS meeting presentation by going to: https://www.aats.org/resources/the-benefit-of-a-novel-biosens-9663.

**Figure:**
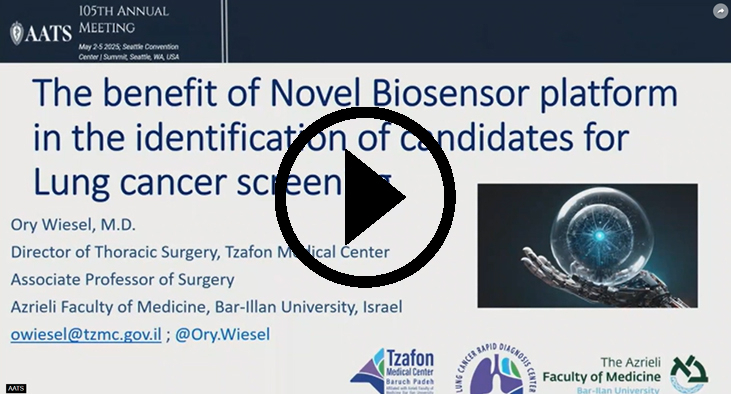

### Audio

You can listen to the discussion audio of this article by going to the supplementary material section below.

## Conflict of Interest Statement

T.S., A.L.A., and M.M.D. are employed by the company EARLY O.M. Ltd. All other authors reported no conflicts of interest.

The *Journal* policy requires editors and reviewers to disclose conflicts of interest and to decline handling or reviewing manuscripts for which they may have a conflict of interest. The editors and reviewers of this article have no conflicts of interest.

## Supplementary Data

### Audio

Audio 1 This is an audio recording of the presentation of the associated abstract and discussion. The abstract presentation ends at 7:32 and the discussion begins at 7:37.

Video 1 Video available at: https://www.jtcvs.org/article/S2666-2736(26)00023-9/fulltext.
